# Mechanistic role of astaxanthin derived from shrimp against certain metabolic disorders

**DOI:** 10.1002/fsn3.2623

**Published:** 2021-11-30

**Authors:** Zubda Yaqoob, Muhammad Sajid Arshad, Muhammad Imran, Haroon Munir, Tahira Batool Qaisrani, Waseem Khalid, Zubia Asghar, Hafiz Ansar Rasul Suleria

**Affiliations:** ^1^ Department of Food Science Faculty of Life Sciences Government College University Faisalabad Pakistan; ^2^ Department of Diet and Nutritional Sciences University of Lahore Lahore Pakistan; ^3^ Department of Agricultural Engineering and Technology Ghazi University Dera Ghazi Khan Pakistan; ^4^ School of Agriculture and Food Faculty of Veterinary and Agricultural Sciences The University of Melbourne Parkville Victoria Australia

**Keywords:** antioxidant, astaxanthin, cardiovascular diseases, diabetes, hypertension, shrimps

## Abstract

Oxidative stress caused by the imbalance between production of oxidants and antioxidants in the body leads to the development of different ailments. The bioactive compounds derived from marine sources are considered to be safe and appropriate to use. Astaxanthin possesses antioxidant activity about 100–500 times higher than other antioxidants such as α‐tocopherol and β‐carotene. It has numerous health benefits and vital pharmacological properties for the treatment of diseases like diabetes, hypertension, cancer, heart disease, ischemia, neurological disorders, and potential role in liver enzyme gamma‐glutamyl transpeptidase which has significance in medicine as a diagnostic marker. The primary source of astaxanthin among crustaceans is shrimps and the presence of astaxanthin protects shrimps from oxidation of polyunsaturated fatty acids and cholesterol. Conclusively, astaxanthin derived from shrimps is very effective against oxidative stress which can lead to certain ailments.

## Graphical abstract

Conclusively, the astaxanthin derived from shrimps is very effective against oxidative stress which become the cause of certain ailments.

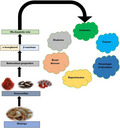

## 1 INTRODUCTION

Metabolic syndrome are a cluster of metabolic disorders in the same individual. The primary agreement concerning the description of the metabolic syndrome was established by the International Diabetes Federation (IDF) in the year of 2005. According to IDF, the main risk factor is obesity which is calculated based on body mass index (BMI) and waist circumference. The additional factors for the diagnosis of metabolic syndrome are reduced high‐density lipoprotein (HDL) cholesterol, fasting plasma glucose, triglycerides, or blood pressure‐. The patho physiology of metabolic syndrome is very composite and is not clearly established. The prevalence of metabolic syndrome is high among those individuals who are older, obese, having insulin resistance, and living a sedentary life style. The main risk factors are excess caloric intake, genetics, weight, lifestyle, and aging (Bonomini et al., 2015).

Metabolic syndrome and the risk factors associated with this syndrome such as atherosclerosis, hypertension, dyslipidemia, and hyperglycemia are the main reasons for increasing medical issues in industrialized countries. The main risk factors for rising metabolic syndrome are physical immobility and a diet high in carbohydrates and fats (O'Neill & O'Driscoll, 2015). Oxidative stress is the main reason that causes metabolic syndrome (Furukawa et al., 2017). Oxidative stress leads elevationed intracellular stage reactive oxygen species (ROS) that causes damage to DNA, proteins, and lipids. Oxidative stress is associated with numerous pathologies (Schieber & Chandel, 2014). Human body is constantly exposed to various types of agents which results in the production of reactive species known as free radicals (ROS/RNS (reactive nitrogen species)) that by relocating of their free unpaired electron caused the oxidation of cells. In order to counter the lethal effects of such activities the human has got endogenous antioxidant systems or it obtains exogenous antioxidants from diet that neutralizes such species and keeps the homeostasis of body (Asmat et  al., 2016).

## 2 OVERVIEW OF ASTAXANTHIN

Astaxanthin is a pinkish‐orange lipophilic carotenoid found in seafoods (crustacean, shells, crab, shrimps, fish), algae, and various plants, giving them their elite colored aspect (Zhao et  al.,  2011). It offers the characteristic reddish pigment to farm‐raised salmon (Ambati et al., 2014). It is a critical carotenoid pigment and a potent antioxidant with confirmed fitness advantages (Khalid & Barrow, 2018). It is documented to have antioxidant activity as a scavenger of free radicals and a quencher of ROS, thereby protecting native molecules (e.g., fatty acids) and cellular membranes from oxidation (Rodrigues et al., 2012). The antioxidant activity of astaxanthin on cells is greater than that of β‐carotene, vitamin C, vitamin E, lutein, lycopene, and other catechins. The antioxidant activity of astaxanthin has been proven to be a 100‐ to 500‐fold greater than α‐tocopherol and 5‐ to 15‐fold greater than that of other carotenoids. Astaxanthin has been shown to inhibit the level of lipid peroxidation, as measured by thiobarbituric acid reactive materials, and increase the level of mobile antioxidants, as measured by glutathione and superoxide dismutase, in rat liver tissues treated with carbon tetrachloride (CCl_4_) (Chen & Kotani, 2016).

## 3 BIO‐AVAILABILITY AND SOURCES OF ASTAXANTHIN

Astaxanthin is biosynthesized by flowers, bacteria, a few fungi, and microalgae. It also can be extracted from crustaceans such as shrimp, crawfish, crabs, and lobster. It represents between 74% and 98% of the entire pigments in crustacean shells (Sila et al., 2015). It is synthesized not nonly by flowers and microorganisms but also by aquatic animals including crustaceans, salmon, and trout (Chintong et al., 2019). It is significantly produced by algal species, especially *Haematococcus pluvialis* (where it accumulates up to 38% at the dry weight foundation), *Chlorella zofingiensis*, *Chlorococcum*, and also by the yeast *Phaffia rhodozyma* (Rao et al., 2013). It confers the wealthy pink color discovered in numerous aquatic species together with the salmonids and crustaceans, and even some nonaquatic species along with the flamingo. Sea creatures cannot produce astaxanthin themselves and is obtained from their diets, which consist of zooplankton and krill. Krill oil contains appreciable amount of astaxanthin (0.1–15 mg/ml) relying on processing methods (Ali‐Nehari et al.,  2011). Shrimp waste is one of the important herbal sources of carotenoids with astaxanthin and its esters as the principal pigments. Several methods for extraction of astaxanthin have been explored. Natural solvent has been used to recover the pigment from crustacean processing discards (Sachindra et al., 2007). Astaxanthin is an effective, but fantastically lipophilic antioxidant, which attributes to health benefits. Astaxanthinm consumption is associated with reduced risk for cancer, cardiovascular diseases, neurodegenerative issues, and other aliments. However, our food lacks astaxanthin as it is specifically found in algae, salmon, and crabs. It is produced through cultivation of the microalga *Haematococcus pluvialis*, which contains about 6% astaxanthin (dry weight). A potato protein‐based delivery is used to test the ability for protecting astaxanthin and enhancing its solubility, bioaccessibility, and bioavailability (Edelman et al., 2019). Bioavailability of astaxanthin can be enhanced by the formation of an ester by conjugating it with succinic anhydride. Astaxanthin succinate diester is more thermally stable and bioavailable to the body tissues than ree astaxanthin (Qiao et al., 2018).

## 4 MECHANISTIC ROLE

Carotenoids are increased by high cholesterol after being absorbed into the body lipids. After being absorbed, astaxanthin makes micelles by mixing with bile acid. Astaxanthin is then incorporated into chylomicron and assimilates with lipoprotein and is then transported to various body tissues to protect different cells against oxidative damages (Komatsu et al., 2017). Astaxanthin stops oxidative damages and reactions by quenching singlet oxygen and free radicals, and it is carried out polyene chain and multiple double bonds present in astaxanthin. Polyene chain has the property of eliminating free radicals from cell membrane (Ambati et al., 2014).

## 5 ANTIOXIDANT POTENTIAL OF ASTAXANTHIN

1,1‐Diphenyl‐2‐picrylhydrazyl is used as a synthetic radical to detect radical scavenging capability of astaxanthin. Astaxanthin has strong antioxidant activity in bio‐membranes. It was previously thought that this activity can be due to conjugated polyene and terminal ring moieties of astaxanthin. Conjugated polyene trap radicals on the membrane surface and terminal ring moieties trap the radicals within the membrane. Also, it was also reported that some astaxanthin and their derivatives have properties of scavenging superoxide anion radical. Superoxide anion radical and hydroxyl radical are considered as highly active ROS. These radicals have been considered as causative agents for arteriosclerosis and ischemic reperfusion injury, and other severe diseases (Hama et al., 2012). Therefore, by scavenging these highly active ROS, it is expected to prevent such ROS‐related diseases. Astaxanthin inhibits the level of lipid peroxidation and increases the level of cellular antioxidants, as measured by thiobarbituric acid reactive substances and glutathione and superoxide dismutase, respectively, in rat liver tissues treated with CCl4 (Chen & Kotani, 2016). The conversion of xanthine dehydrogenase to xanthine oxidase and protein carbonyl level in rat liver tissues were inhibited by astaxanthin in a severe oxidative condition (Curek et al., 2010). Astaxanthin also induces the expression of nuclear factor‐erythroid 2‐related factor 2 mRNA in mouse liver (Yang et al., 2014).

## 6 ASTAXANTHIN AS AN ANTIMICROBIAL AGENT

The extracts of astaxanthin showed better antibacterial activity aganist several organisms, such as *Bacillus subtilis, Salmonella typhi, Staphylococcus aureus,* and *Pseudomonas aeruginosa* than the standard chloramphenicol. Among all, *Pseudomonas aeroginasa* showed maximum inhibition (Ushakumari & Ramanujan, 2013). Both minimum bactericidal concentration and minimal inhibitory concentration methods confirmed significant antibacterial activity (Shanmugapriya et al., 2018). A study conducted on the *Sphingomonas faeni* methanol extract showed that the extract had antibacterial activity against *Psychrobacter, Rhodococcus, Arthrobacter, Pseudomonas, Leuconostoc,* and *Sphingomonas* strains (Mageswari et al., 2015). Another study conducted shows that, during wound healing process in animals, a biofilm is produced with astaxanthin which reduces the antibacterial activity or bacterial growth (Weintraub et al., 2017).

## 7 ASTAXANTHIN EFFECT ON CARDIOVASCULAR DISEASES

Anti‐inflammatory activity of astaxanthin was both studied and examined in human subjects and experimental animals. Oxidative stress and inflammation are the pathophysiological factors of atherosclerotic cardiovascular disease and astaxanthin is considered as a therapeutic agent against this disease. (Fassett & Coombes, 2011). Astaxanthin protects the myocardium in form of disodium succinate astaxanthin (DDA) in animals. Myocardial ischemia reperfusion model was used to access effectiveness of DDA. The results showed that after 4 days of pretreatment with DDA at 25, 50, and 75 mg/kg body weight in Sprague–Dawley rats, myocardial infarct size was reduced and also myocardial salvage improve (Ambati et al., 2014). When the myocardial tissues of rat were pretreated with 150 and 500 mg kg^−1^ day^−1^ dosage of DDA for 7 days, astaxanthin was found in myocardial tissues. Among the trated rats, a large number of number of rats like normotensive Wistar Kyoto rats (NWKR), stroke prone spontaneously hypertensive rats (SPSHR), and hypertensive rats showed an effect on blood pressure, (SHR; Monroy‐Ruiz et al., 2011). Another study of astaxanthin on rats showed that the among the two groups one which was fed with 0.08% astaxanthin showed higher heart mitochondrial membrane potential and contractility index than the control group (Nakao et al., 2010). Dos of 100 mg and 500 mg/100 g of astaxanthin in hypercholesterolemic rabbits revealed that astaxanthin prevented the activities of enzymes, paraoxonase, thioredoxin reductase activities, oxidative stress parameters, and lipid profile from hypercholesterolemia‐induced protein oxidation (Augusti et al., 2012). Treatment of astaxanthin also showed decreased peroxynitrite levels and increased nitric oxide levels in human umbilical vein endothelial cells and platelets (Khan et al., 2010). The health perspectives of astaxanthin are shown in Table 1.

**TABLE 1:** Health perspectives of Astaxanthin

Disorder	Mechanism	References
Anticancer	Suppressed the proliferation by interrupting cell cycle progression	Kim et al., 2016
	Inhibited the phosphorylation of ERK and the enhanced expression of p27^kip−1^	
	Suppressed cellular growth by inhibiting the action of 5‐α‐reductase	Kurihara et al., 2002
	Inhibited lipid peroxidation	
	Inhibited tumor growth and lowered proliferation rate	Ni et al., 2017
	Increased cleaved caspase−3 and apoptotic cells	
	Downregulated the expression of STAT−3 target genes	Kowshik et al., 2014
	Reduced microvascular density, thereby preventing tumor progression	
	Prevented the development and progression of HBP carcinomas	
	Inhibited JAK−2/STAT−3 signaling	
	Inhibited the proliferation of H22 cells, promoted cell necrosis, and induced cell cycle arrest in G2	Shao et al., 2016
	Induced LX−2 cells apoptosis which may be by regulating miR−29b	Zhu et al., 2019
	Inhibited Bcl−2 expression levels and elevated Bax and Caspase−3 expression levels	
Anti‐inflammatory	Inhibited expression or production of inflammatory mediators and cytokines	Bolin et al.2010
	Suppressed the activation of nuclear factor‐kB	
	Inhibited the expression of inducible nitric oxide synthase and cyclooxygenase−2	Choi et al., 2008
	Suppressed the expression of the scavenger receptors SR‐A and CD36) the activity	Kishimoto et al., 2010
	Inhibited the expression of MMPs and mRNA	
	Suppressed the inflammatory mediators, that is, interleukin (IL)−1β, inducible nitric oxide synthase (iNOS), and COX−2	
	Reduced the production of proinflammatory cytokines (TNF‐α and IL−6)	Macedo et al., 2010
	Enhanced neutrophil phagocytic and microbicidal capacity	
	Suppressed superoxide anion and hydrogen peroxide production	
	Inhibited macrophage inflammation	Lee et al., 2003
	Suppressed the proinflammatory cytokines, prostaglandins, and NO	
	Suppressed IκB‐dependent NF‐κB activation	
Antimicrobial	Upregulated the interferon gamma (IFN‐γ), and increased interleukin 2 (IL−2) and IL−10	Davinelli et al., 2019
	Showed antibacterial activity against strains of *Psychrobacter, Rhodococcus, Arthrobacter, Pseudomonas, Leuconostoc,* and *Sphingomonas*	(Mageswariet al., 2015).
Bone health	Suppressed the enhancement of serum calcium, inorganic phosphorus, alkaline phosphatase, total cholesterol, and tartrate‐resistant acid phosphatase (TRAP) activity	Hwang et al., 2018
	Inhibited osteoclast formation through the expression of the nuclear factor of activated T cells (NFAT) c1, dendritic cell‐specific transmembrane protein (DC‐STAMP), TRAP, and cathepsin K	
	Decreased the osteoclast number and increase the osteoblast number	Balci et al., 2018
	Suppressed the expression of NFATc1 protein	Lelovas et al., 2008
	Lowered the expression of MMP−1, MMP−3, and MMP−13	Chen et al., 2014
	Reduced the phosphorylation of chondrocytes induced by MAPK p38, ERK1/2, and IL−1beta	
	Increased the proliferation and differentiation of osteoblasts	Kim et al., 2010
Cardioprotective	Inhibited low‐density lipoprotein oxidation, and increased high‐density lipoprotein cholesterol and adiponectin levels	Kishimoto, 2016
	Suppression of oxidative stress and improvement of cardiac contractility	Kato et al., 2020
	Improved the respiration of rat heart mitochondria	Krestinina et al., 2020
	Decreased the level of cyclophilin D	
	Increased the level of adenine nucleotide translocase	
Blood glucose and antiobesity	Prevented the destruction of pancreatic β‐cells [74].	Ikeuchi et al., 2007
	Decreased the HFFD‐induced activation of serine kinases (JNK and ERK)	Uchiyama et al., 2002
	Increased fatty acid utilization	
	Induced uncoupling protein 1 (UCP1) in mitochondria	Gammone & D'Orazio, 2015
	Activated the hepatic IRS‐PI3K‐Akt signaling pathway and improved glucose metabolism	Arunkumar et al., 2012
	Normalized the activities of hexokinase, pyruvate kinase, glucose−6‐phosphatase, fructose−1,6‐bisphosphatase, and glycogen phosphorylase	Bhuvaneswari & Anuradha, 2012

## 8 ASTAXANTHIN ON STRESS

Astaxanthin not only reduces and maintains the oxidative stress, which occur physiologically in mitochondria, but also increases mitochondrial oxygen utilization and prevents loss of mitochondrial membrane potential (MMP). Astaxanthin performs all these functions even after stimulation with H_2_O_2_. It proved that mitochondrial functions are sustained due to astaxanthin which protects mitochondrial redox balance (Kuroki et al., 2013; Wolf et al., 2010; Zhang et al., 2016). These findings suggested that astaxanthin can maintain mitochondrial integrity, prevent mitochondrial dysfunction, and reduce oxidative stress. In a in vitro model of inflammatory preeclampsia, astaxanthin was observed to enhance MMP, reduce ROS levels, and prevent heat stress‐induced impairment of blastocyst development (Xuan et al., 2016). Comparatively in an in vivo study, in geriatric dogs treated with mitochondrial function got restored by mitigating the oxidative damage. This study showed that increased ATP production, mitochondrial content, and respiratory chain complex activity and proved that increasing mitochondrial efficiency can prevents aging (Park et al., 2013). A study carried on mitochondria of ischemic mice suggests that treatment of astaxanthin on mitochondria, isolated from the myocardium, reduces mitochondrial ROS production, mitochondria depolarization, and swelling (Pongkan  et al., 2017 ).

## 9 ASTAXANTHIN ON LIPID PROFILE

It is stated that the changes in lipid profile after astaxanthin supplementation in a in vitro membrane model was observed that of lutein and β‐carotene. Results obtained showed that astaxanthin preserved the membrane consistency by inhibiting the formation of lipid peroxide when compared with lutein and β‐carotene which not only damaged the structure of the membrane but also raises lipid hydroperoxide levels (Ursoniu et al., 2015). Astaxanthin also acts as a peroxisome proliferator‐activated receptor γ (PPAR‐γ) agonist and PPAR α agonist, which reduces cellular lipid accumulation in lipid‐loaded hepatocytes (Jia et al., 2012). During initialization of mitochondrial aerobic metabolism, astaxanthin takes up increased amount of peroxisome proliferator‐activated receptor‐γ coactivator 1‐α (PGC‐1α) and increases the usage of lipid in skeletal muscle (Liu et al., 2014). A study conducted on obese mice showed that astaxanthin, being an efficient antioxidant, also decreases abdominal fat‐pad weight and liver weight (Ursoniu et al., 2015). Another 12‐week study was conducted on 61 nonobese Japanese subjects who had 120–200 mg/dl of fasting serum triglyceride (moderate hypertriglyceridemia). Results showed that astaxanthin doses of 12 and 18 mg/day decreased the serum triglyceride levels comparatively and doses of 6 and 12 mg/day increased serum HDL cholesterol (Yoshida et al., 2010).

## 10 ASTAXANTHIN ON SKIN HEALTH

Daily consumption of natural astaxanthin has many benefits for skin. Daily consumption of 4 mg astaxanthin within 2 months can lead to better health and quality of skin with good appearance. Astaxanthin also protects the skin from damages caused by exposure to ultraviolet rays. Clinical trial was carried out in the United States in 2006 on 46 middle‐aged women. The subjects were divided into two groups one taking placebo and other taking 4 mg of astaxanthin per day. Results measured and collected from dermatological devices, which includes assessment by a dermatologist, showed that natural astaxanthin enhances skin quality and beauty by fighting wrinkles, improving skin elasticity, maintaining youthful appearance, increasing skin moisture levels, and reducing visible signs of aging due to UV. All these effects appeared within 4–6 weeks of trial (Yamashita, 2005). Another clinical open‐label study performed on 30 healthy females showed improvement in skin condition after daily dose of 6 mg oral supplementation and 2 ml (78.9 μM) solution of natural astaxanthin. Results, collected from dermatological devices, showed improved skin condition involving improvement in age spot size, elasticity, wrinkles, and skin texture. Improvement in moisture content of the corneocyte layer was also observed. These results conclude that by having combination of oral supplements and topical treatment of natural astaxanthin can improve skin condition in all layers including the basal layer, the dermis, the epidermis, and corneocyte layer (Tominaga et al., 2012). Among many other health‐associated problems, aging also causes skin pigmentation (age spots). A study conducted on skin pigmentation in Japan revealed that astaxanthin inhibits the stem cell factor‐associated stimulation of pigmentation. This function performed by astaxanthin is dose‐dependent, higher the dose the more pigmentation inhibition will be (Nakajima et al., 2012).

## 11 ASTAXANTHIN ON DIABETES MELLITUS

The risk of diabetes increases due to hyperglycemia and other metabolic syndromes which also stimulate ROS production in mitochondria (Kim & Kim, 2018). Oxidative stress causes chronic inflammatory and increased level of ROS production which results in apoptosis in the liver, endothelium, pancreas, kidney, and also causes cellular dysfunction (Roohbakhsh et al., 2017). A study was conducted to evaluate the hypoglycemic effect on astaxanthinin alloxan‐induced diabetic and normal mice, and plasma glucose levels were examined. Results obtained were compared with metformin and gliclazide and showed significant decrease in plasma glucose in alloxan‐induced diabetic mice, which were administered with astaxanthin in doses of 5 and 10 mg/kg, while normal mice showed slight decrease due to suppression of postprandial hyperglycemia by oral administration of astaxanthin. (Wang et al., 2012).

## 12 ASTAXANTHIN AS ANTICANCER AGENT

Initially, practices consuming diet rich in carotenoids for prevention of cancer and reducing the effect of carcinogenesis proved that diet is one of the main factors in adjusting and modulating the effect of certain tumor developments (Barros et al., 2012). In a 1‐year trial, patients having squamous cell carcinoma of the head and neck were treated with astaxanthin and results proved that astaxanthin has the to prevent the development of new cases (Al‐Bulishi et al., 2015). Astaxanthin also showed its antitumor efficacy in studies conducted on various in vitro and in vivo cancer models, proving itself as a chemotherapeutic agent. Being an excellent antioxidant, astaxanthin is also a good therapeutic agent for various diseases and causes no side effects like toxicity (Zhang & Wang, 2015).

## 13 ASTAXANTHIN ON LIVER HEALTH

The effect of astaxanthin on oxidative stress, hepatic damage, inflammatory cells infiltration, iron deposition, and prevention of early development of fibrosis was observed in CCl4‐induced rats. After the CCl_4_ administration in rats, increased activities of aspartate aminotransferase, alanine aminotransferase, and alkaline phosphatase were observed when compared to normal rats. These changes were reduced and were brought to normal with treated with astaxanthin. Astaxanthin also lowered myeloperoxidase (MPO) activity and restored the catalase and superoxide dismutase activities. Further investigation also revealed that astaxanthin prevented the infiltration of inflammatory cells in the liver of CCl_4_‐administered rats. It also prevented decreased free iron deposition and fibrosis. Astaxanthin plays an important role in improvement of hyperlipidemia, preventing liver damage due to oxides, and retaining proper functioning of liver. Antioxidative effects of astaxanthin prevent liver pathologies (Chen & Kotani, 2016).

## 14 ASTAXANTHIN ON BRAIN HEALTH

Being an antioxidant, astaxanthin provides health benefits to the brain beyond blood–brain barrier. Astaxanthin can prevent mitochondrial abnormalities, 6‐hydroxydopamine‐induced neuronal apoptosis, and reactive oxygen generation within SH‐SY5Y cells. Due to these properties of astaxanthin, it is used for testing number of neurological diseases in mammals (Liu & Osawa, 2009). Astaxanthin protects PC12 cells against damages generated by β‐amyloid peptide 25–35; thus its performs its functions in early stages of adjuvant therapy in Alzheimer's disease and is a potential neuron protectant (Chang et al., 2010). A research performed on young adult rat brain shows that astaxanthin might antagonize the ethanol‐induced facilitation and its antioxidant properties might be involved in cortical spreading depression propagation (Grimmig et al., 2017).

When 24 h reperfusion and 2 h of middle cerebral artery occlusion (MCAO) was performed on rats, results showed that astaxanthin has ability to prevent cerebral ischemic injury. Pretreating rats with astaxanthin, twice in 1 and 5 h intragastrically, showed improvement in neurological deficit and decreasing in infarct volume. The efficacy of results depends on dosage (Lu et al., 2010).

## 15 ASTAXANTHIN ON BONE HEALTH

Aging brings number of health disorders with itself, CVDs, cancer, arthritis, type 2 diabetes, and many more. Bone health problem is one of the health disorders which increases with aging. Osteoarthritis is the common bone health disorder, which is associated with soft and porous bones, mainly due to wearing down of cartilage at the end of bones, leading to lower bone mass and weaker joints. Astaxanthin, as an antioxidant, can prevent this bone health disorder by suppressing the oxidative stress and NrF2 signaling pathway (Sun et al., 2019).

## 16 ASTAXANTHIN AS AN ANTIOBESITY AGENT

Obesity is the worldwide malnutrition health problem which leads to many other disorders, including hypertension, hyperlipidemia, type 2 diabetes, and cardiovascular diseases (CVDs). Antiobesity effect of astaxanthin led to the development of safe antiobesity agents by the researchers. (Fakhri et al., 2018). Astaxanthin serves as an antiobesity agent by reducing plasma cholesterol, preventing weight gain, increasing hepatic expression of endogenous antioxidant genes, decreasing plasma and liver triacylglycerol, decreasing MPO and nitric oxide synthases, and by making splenocytes less sensitive to lipopolysaccharide stimulation. It is thought that astaxanthin can prevent metabolic disturbances and inflammation that are related to obesity (Bhuvaneswariet al., 2010; Yang et al., 2014). A study showed that astaxanthin has an important role in increasing lipid usage during exercise. It also culminates modified muscular metabolism and superior physical function, resulting in decreased body fat and increased muscular action improvement during exercise (Aoi et al., 2014). Astaxanthin gives improved effects on obesity and insulin resistance by acting as an antagonist or agonist in the form of novel selective PPAR‐γ modulator (Inoue et al., 2012).

## 17 CONCLUSIONS

Bioactive compounds from natural sources are gaining popularity nowadays. Astaxanthin is a rich source of natural carotenoid pigment and potent antioxidant. Astaxanthin has higher antioxidant activity than β‐carotene and vitamin C. The microalga *Haematococcus pluvialis* is the richest source of astaxanthin because it has highest dry weight content about 6%. Astaxanthin consumption is associated with reduced risk for cancer, cardiovascular diseases, and neurodegenerative issues. Astaxanthin has various properties such as anti‐inflammatory, antioxidative activity, antiobesity properties, immunomodulatory activity, protection, and safety of its usage which shows the higher interest in astaxanthin amount on the part of various scientific centers and food manufacturers. Various studies depicted that astaxanthin has useful effects on the human body. Further research is needed to extend the knowledge and consciousness about astaxanthin. Some analyses are still required to elucidate the process behind the effectiveness of astaxanthin.

## AUTHOR CONTRIBUTIONS

**Zubda Yaqoob:** Writing‐original draft (equal). **Muhammad Sajid Arshad:** Conceptualization (lead); Project administration (lead); Supervision (lead). **Muhammad Imran:** Conceptualization (equal). **Haroon Munir:** Investigation (equal). **Tahira Batool Qaisrani:** Writing‐review & editing (supporting). **Waseem Khalid:** Writing‐review & editing (supporting). **Zubia Asghar:** Writing‐review & editing (supporting). **Hafiz Suleria:** Writing‐review & editing (equal).

## Data Availability

Data sharing is not applicable to this article as no datasets were generated or analyzed during the current study.
